# Bizarre parosteal osteochondromatous proliferation in the lingual area of the mandibular body versus osteochondroma at the mandibular condyle

**DOI:** 10.1186/s12957-016-0777-9

**Published:** 2016-02-11

**Authors:** Soung Min Kim, Hoon Myoung, Sang Shin Lee, Yeon Sook Kim, Suk Keun Lee

**Affiliations:** Department of Oral and Maxillofacial Surgery, Dental Research Institute, School of Dentistry, Seoul National University, Seoul, Korea; Department of Dental Hygiene, Cheongju University, Cheongju, Korea; Department of Oral Pathology, College of Dentistry, Gangneung-Wonju National University, 123 Chibyun-dong, Gangneung, Korea

**Keywords:** Bizarre parosteal osteochondromatous proliferation, Osteochondroma, Immunohistochemistry

## Abstract

**Background:**

Bizarre parosteal osteochondromatous proliferation (BPOP) is benign and usually occurs in the small tubular bones of the hands and feet, but it is extremely rare in the oral and maxillofacial region.

**Methods:**

The present study compares a case of BPOP occurring in the lingual area of the right mandibular body with a representative case of osteochondroma occurring in the left mandibular condyle using immunohistochemical methods.

**Results:**

BPOP showed no continuity to the cortical bone of the mandible on X-ray and was histologically composed of immature cartilage and bone tissues, whereas osteochondroma showed overgrowth of hypertrophic chondrocytes accompanied by mature bone with endochondral ossification. Although BPOP showed no features of cellular atypia or malignant transformation, it expressed more osteogenic proteins, including BMP-2, BMP-4, RUNX2, OC, AP, OPG, RANKL, CTGF, and bFGF, than osteochondroma. Furthermore, the perichondral spindle cells and marrow osteoblasts/fibroblasts of BPOP showed stronger immunoreaction of PCNA, p53, β-catenin, BCL2, pAKT, survivin, 14-3-3, CEA, EMA, pan-K, and S-100 than the tumor cells of osteochondroma.

**Conclusions:**

Therefore, it was presumed that similar to embryonal osteochondroid tissue, BPOP might be activated by osteogenic and oncogenic signaling and that this increased signaling may explain the rapid growth and high recurrence of BPOP.

## Background

Bizarre parosteal osteochondromatous proliferation (BPOP) is a rare, benign osteocartilaginous lesion which was first described in 35 lesions from the small bones of the hands and feet by Nora et al. in 1983 [1]. Many cases involving various other sites, including the zygoma, maxilla, and mandible, have been reported [2–5]. BPOP usually shows key radiological features, including lack of histological features characterized by three distinct components with variable degrees of representation: (1) hypercellular cartilage with calcification and ossification, with the calcified cartilage having a characteristic basophilic tinctorial quality; (2) cancellous bone undergoing maturation; and (3) spindle cell stroma without cytologic atypia [6–9]. BPOP, also known as Nora’s lesion, often behaves like a malignant tumor, clinically and microscopically [10]. Histological features of BPOP include hypercellular spindle cells, a blue tinctorial quality in the osteocartilaginous interfaces, and a scattering of binucleated or bizarre enlarged chondrocytes [11].

Generally, it is presumed that BPOP arises from periosteal tissues through a process of cartilaginous metaplasia. BPOP can be easily confused, both clinically and microscopically, with other benign and malignant lesions of the bone, including osteochondroma, myositis ossificans, florid reactive periostitis, turret exostosis, parosteal osteosarcoma, and chondrosarcoma [12, 13]. BPOP has been reported to have a high rate of recurrence (about 50 %) but without metastasis [9, 14]. Translocation between chromosomes 1 and 2, t(1;17)(q32;q21), and inversion of chromosome 7, inv(7)(q22q32), have been observed in BPOP [15–17].

Osteochondroma is the most common tumor of the skeletal bones, but it is relatively uncommon in the jaws at the condyle or the tip of the coronoid process [18]. This benign cartilage-capped tumor simulating unilateral condylar hyperplasia is usually discovered incidentally on radiographic examination or on palpation of a protruding mass in the affected area [19]. Malocclusion and progressive facial asymmetry are common findings in most cases of condylar osteochondroma [20]. Computed tomography and magnetic resonance imaging depicted the central part of the exophytic bone lesion as having continuity to the underlying bone marrow, which is considered to be the typical finding of osteochondroma compared to BPOP [21, 22]. In addition, an inversion of chromosome 7 [inv (7)(q22q32)] has also been observed in osteochondroma [23].

The present study used immunohistochemical (IHC) staining using different antisera to compare a case of BPOP occurring at the lingual area of the right mandibular body to a representative case of osteochondroma occurring in the left mandibular condyle.

## Methods

### A case of BPOP and osteochondroma

**Fig. 1:**
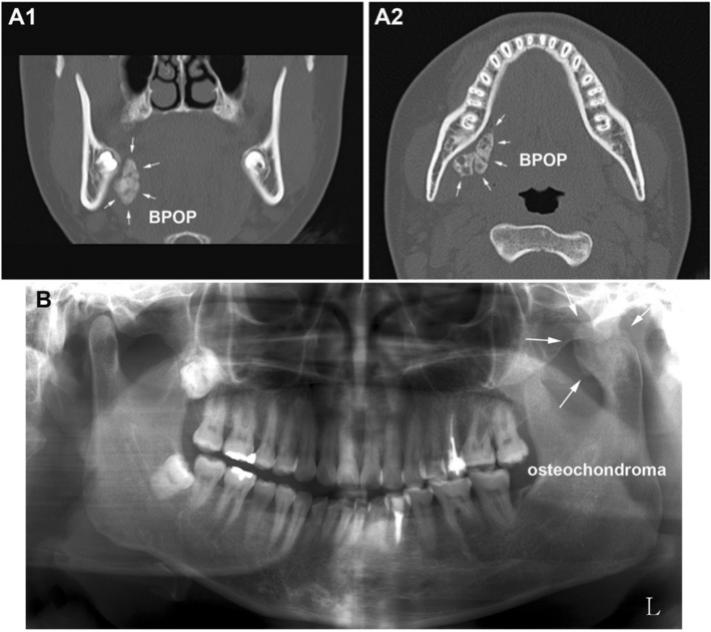
Radiographic views of this study. **A** Computed tomography of BPOP, an irregularly calcified mass located on the lingual side of the mandibular body without cortical attachment (*arrows*). **A1** Frontal plane. **A2** Horizontal plane. **B** Panoramic view of osteochondroma, enlarged left condylar head (*arrows*)

**Fig. 2:**
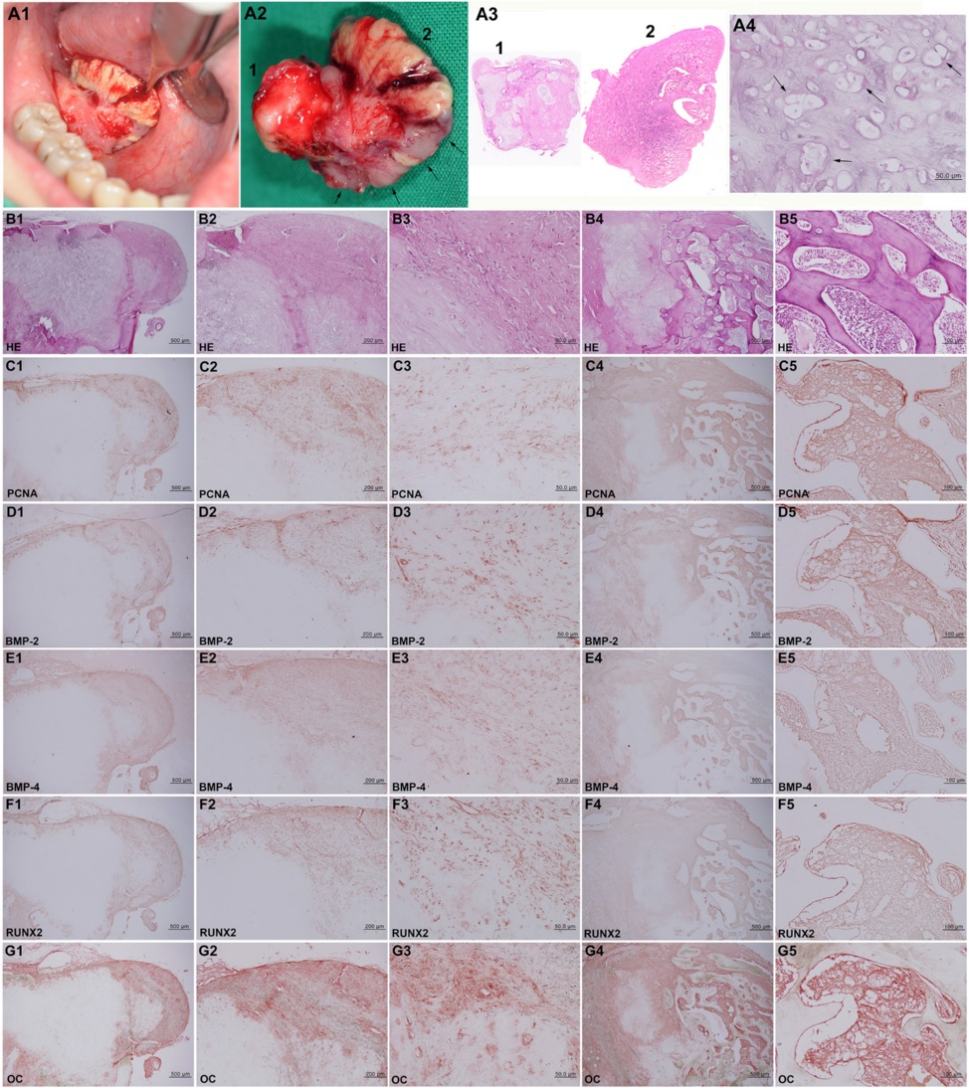
BPOP. **A1** Sublingual ulceration with a whitish calcified mass. **A2** Removed mass showing partial bluish color (*arrows*). **A3** BPOP specimen was composed of cartilaginous (*1*) and osseous (*2*) tissue. **A4** Bizarre chondrocytes (*arrows*) in cartilaginous tissue. **B** HE stain. **B1**–**B3** (area *1* of **A3**). **B4**, **B5** (area *2* of **A3**). **B1** and **B3** show core cartilage covered with thick perichondral fibrous tissue. **B4** and **B5** show anastomosing trabecular bone centered from cartilaginous tissue, mimicking endochondral ossification. **C**–**G** IHC stains with no background stain. **C** PCNA. **D** BMP-2. **E** BMP-4. **F** RUNX2. **G** OC

A 17-year-old female noticed a hard sublingual mass 3 months prior and experienced stiffness during yawning. The oral mucosa then became ulcerated, and a calcified mass was exposed with whitish and partially bluish color. On computed tomography, the mass was ovoid and lobulated with irregular calcification and measured about 30 × 20 mm on the lingual area of the right mandibular body. The calcified mass showed no continuity with the cortical bone of the mandible on X-ray (Fig. 1A); therefore, it was enucleated through simple intraoral dissection at Seoul National University Dental Hospital. The removed specimen was submitted to the Department of Oral Pathology, Gangneung-Wonju National University Dental Hospital (GWNUDH) for analysis. It was composed of cartilaginous and osseous tissues on gross observation. The cartilaginous tissue was approximated to the lingual surface of the mandibular body, while the osseous tissue had grown toward the sublingual area (Fig. 2A). The lesion was diagnosed as BPOP through pathological examination (OS2014-25).

A 39-year-old female presented with severe malocclusion and facial asymmetry, which had slowly progressed for 4 years. She was referred to GWNUDH with a chief complaint of slight pain on the left temporomandibular joint during mouth opening. Her left mandibular condyle was severely enlarged with cortico-medullary continuity from adjacent bone structures on orthopantomogram (Fig. 1B). The tumorous condyle was surgically removed by high condylectomy and diagnosed as osteochondroma through pathological examination (S2014-4).

### Immunohistochemical study

The removed specimens of BPOP and osteochondroma were separately fixed in 10 % neutral formalin, decalcified with 5 % nitric acid, embedded with paraffin, and sectioned to 4-μm thickness. The microsections were routinely stained with hematoxylin and eosin (HE) and immunohistochemical (IHC) staining using the following antisera: proliferating cell nuclear antigen (PCNA; Santa Cruz Biotech, USA), bone morphogenetic protein (BMP)-2 (Santa Cruz Biotech), BMP-4 (Santa Cruz Biotech), runt-related transcription factor 2 (RUNX2; Abcam, Cambridge, UK), osteocalcin (OC; DAKO, Denmark), alkaline phosphatase (AP; DAKO), osteoprotegerin (OPG; Santa Cruz Biotech), receptor activator of nuclear factor-kappaB ligand (RANKL; Santa Cruz Biotech), connective tissue growth factor (CTGF; Abcam), basic fibroblast growth factor (bFGF; DAKO), p53 (Santa Cruz Biotech), v-akt murine thymoma viral oncogene homologue 1 (Santa Cruz Biotech), phosphorylated at Thr 308 (pAKT), β-catenin (Santa Cruz Biotech), B cell lymphoma 2 (BCL2; Santa Cruz Biotech), 14-3-3 (Santa Cruz Biotech), survivin (Santa Cruz Biotech), carcinoembryonic antigen (CEA; Santa Cruz Biotech), epithelial membrane antigen (EMA; Abcam), pan-keratins (pan-K; Santa Cruz Biotech), and S-100 (Santa Cruz Biotech, USA). IHC staining was performed using the indirect triple sandwich method [24, 25]. Our institutional review board approved the examination of these biopsy specimens by the Department of Oral Pathology, GWNUDH (IRB 2015-07).

## Results

On histological observation, the BPOP specimen disclosed inner cartilaginous tissue and outer osseous tissue (Fig. 2A3). The cartilaginous tissue was immature and contained many bizarre proliferating chondrocytes (Fig. 2A4) with multifocally distributed perichondral fibrous tissue mainly composed of spindle cells (Fig. 2B1–B3). The osseous tissue showed linearly elongated trabecular bones that stemmed from the cartilaginous tissue (Fig. 2A3). The endochondral ossification was almost abortive but extensively produced trabecular bones (Fig. 2B4). There was no atypical cellular change; however, the perichondral fibrous tissue was thickened with proliferating spindle cells.

Osteochondroma, on the other hand, showed exophytic growth of cartilaginous tissue, followed by trabecular bone ossification at the mandibular condyle. The condylar cartilage almost entirely lacked perichondral fibrous tissue but contained a thick layer of aggregated and proliferative hypertrophic chondrocytes (Fig. 5A). Endochondral ossification was evident with features of chondroid tissue embedded in ossifying bony tissue (Fig. 5B).

**Fig. 3:**
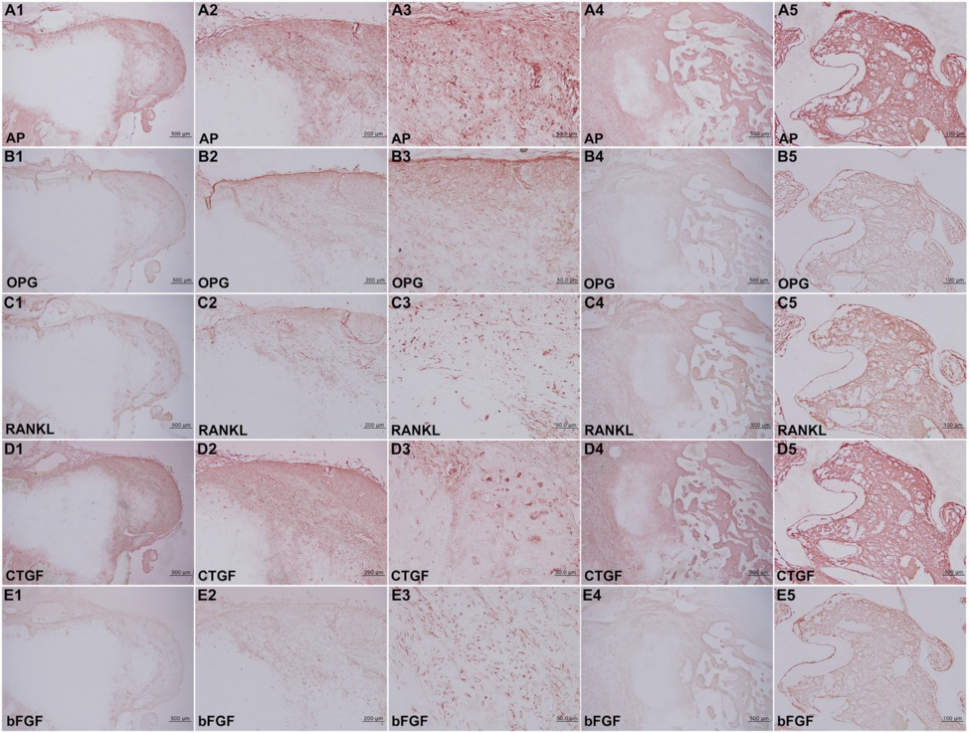
BPOP. IHC stains with no background stain. **A** AP. **B** OPG. **C** RANKL. **D** CTGF. **E** bFGF. (*1*–*3*: cartilaginous tissue of Fig. 2A3. *4*–*5*: osseous tissue of Fig. 2A3)

IHC staining for osteogenetic protein expression in the BPOP specimen was conspicuously positive for both BMP-2 and BMP-4, which are known to indicate osteogenesis and chondrogenesis, respectively, while osteochondroma was much more weakly positive for these proteins. Particularly, the spindle cells of the perichondral fibrous tissue and marrow osteoblasts and fibroblasts of BPOP were strongly positive for OC, AP, and CTGF and consistently positive for OPG, RANKL, and bFGF (Figs. 2D–G and 3), whereas the proliferating chondrocytes and osteoblasts of osteochondroma (Fig. 5A, B) were weakly positive for BMP-2, BMP-4, OC, AP, OPG, CTGF, and bFGF and rarely positive for RUNX2 and RANKL (Fig. 5D–L).

Regarding the proliferative activity observed by PCNA immunoreaction, the BPOP specimen showed a conspicuous positive reaction in the spinous cells of the perichondral fibrous tissue and in some osteoblasts/fibroblasts of the osseous tissue (Fig. 2C), while osteochondroma was rarely positive (Fig. 5C).

**Fig. 4:**
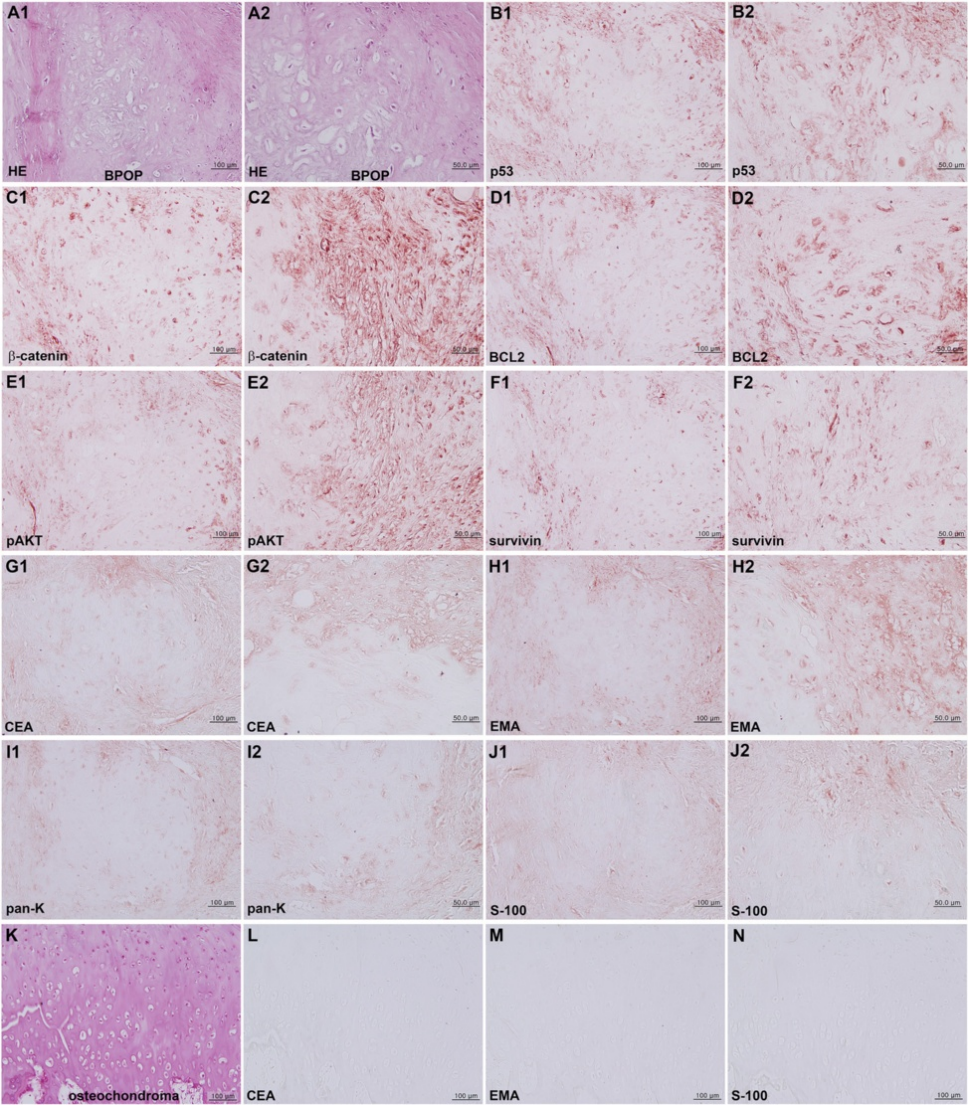
**A–J** BPOP. **A** HE stain, noted bizarre chondrocytes without cellular atypia. **B–J** IHC stains with no background stain. **B** p53. **C** β-catenin. **D** BCL2. **E** pAKT. **F** Survivin. **G** CEA. **H** EMA. **I** pan-K. **J** S-100. **K–N** Osteochondroma. **K** HE stain noted the tumorous growth of chondrocytes. **L** CEA. **M** EMA. **N** S-100.

**Fig. 5:**
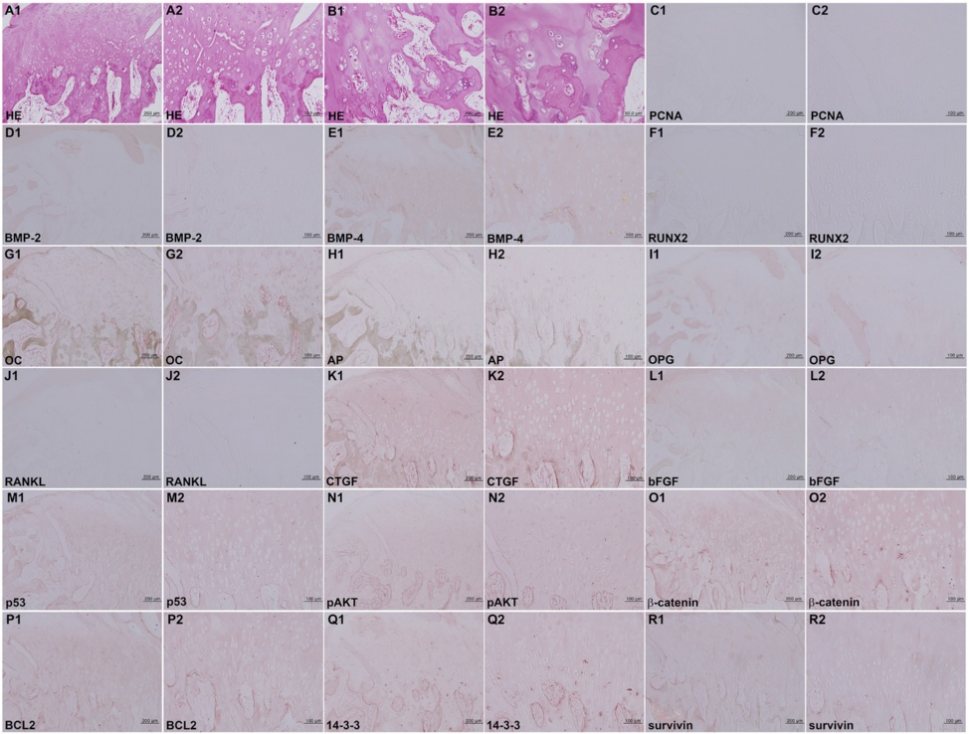
Osteochondroma. **A, B** HE stain noted hyperplastic chondrocytes undergoing abortive endochondral ossification. **C–R** IHC stains with no background stain. **C** PCNA. **D** BMP-2. **E** BMP-4. **F** RUNX2. **G** OC. **H** AP. **I** OPG. **J** RANKL. **K** CTGF. **L** bFGF. **M** p53. **N** pAKT. **O** β-catenin. **P** BCL2. **Q** 14-3-3. **R** Survivin.

On IHC staining of oncogenic protein expression, the bizarre chondrocytes of BPOP were strongly positive for β-catenin, BCL2, and pAKT and consistently positive for p53 and survivin (Fig. 4B–F), while some tumor cells of osteochondroma were positive for β-catenin and 14-3-3 and weakly positive for p53, pAKT, BCL2, and survivin (Fig. 5M–R).

On the other hand, the biomarkers of chondrosarcoma, such as CEA, EMA, pan-K, and S-100 [26–28], showed a slight positive reaction in the spindle cells of perichondral fibrous tissue (Fig. 4G–J) but an almost negative reaction in the tumor cells of osteochondroma (Fig. 4L–N).

## Discussion

On histological observation of the present cases, osteochondroma showed increased endochondral ossification with overgrowth of hypertrophic chondrocytes, while BPOP showed abortive endochondral ossification. The immature cartilaginous tissue of BPOP abruptly produced the trabecular bones that were elongated linearly in a radiating fashion from the core cartilage. While the hypertrophic chondrocytes of osteochondroma produced trabecular bones that were subsequently connected with the original bone marrow, the cartilaginous tissue of BPOP, probably derived from the parosteal mesenchyme, produced trabecular bones in the external direction from the core cartilage, resulting in marrow discontinuity between BPOP and the original bone. However, the trabecular bone growth of BPOP was more active and extensive than that of osteochondroma, and BPOP had multiple cartilaginous tissues which were immature and diffusely scattered, while osteochondroma had a thick cartilage cap on the condylar head. These findings directly indicate that BPOP may have higher recurrence rate than osteochondroma.

In the IHC stains, BPOP showed more intense expression of osteogenic proteins (BMP-2, BMP-4, RUNX2, OC, AP, OPG, RANKL, CTGF, and bFGF) than the ostoeochondroma. BMP-2 and BMP-4, which are markers of osteogenesis and chondrogenesis, respectively, were consistently positive in BPOP but only weakly positive in osteochondroma. Furthermore, the biomarkers of ossification (RUNX2, OC, AP, OPG, and RANKL) and the biomarkers of mesenchymal growth (bFGF and CTGF) were much stronger in BPOP than in osteochondroma. It was thought that BPOP grew more actively than osteochondroma by producing immature cartilage and bone.

The higher expression of osteogenic proteins in BPOP than in osteochondroma was coincident with increased expression of PCNA in the perichondral spinous cells and marrow osteogenic cells of BPOP. Osteochondroma was rarely positive for PCNA. The present study also performed IHC examination for different oncogenic proteins (p53, β-catenin, BCL2, pAKT, and survivin) and the biomarkers of chondrosarcoma (CEA, EMA, pan-K, and S-100) in BPOP and osteochondroma. For the surgical approach for the osteochondroma, not like that for BPOP, several factors, such as complete excision with condylar reconstruction, mandibular contouring, and reconstruction of normal occlusion, must be considered. Virtual surgical simulation for guidance of excision of the mandibular condyle and combined correction of dentofacial deformities can be recommended recently [29].

The oncogenic protein expression of perichondral spindle cells and marrow osteoblasts/fibroblasts of BPOP was strongly positive for p53, β-catenin, BCL2, pAKT, and survivin, while osteochondroma was weakly positive for β-catenin and 14-3-3 and rarely positive for p53, BCL2, pAKT, and survivin. Furthermore, BPOP was consistently positive for CEA, EMA, pan-K, and S-100, while osteochondroma was rarely positive for these proteins.

Immunohistochemically, BPOP showed the expression of bFGF and vascular endothelial growth factor (VEGF), similar to those occurring in endochondral ossification in the growth plate. Thus, BPOP is considered as a reparative process which is occasionally confused with other benign or malignant conditions [30]. It was presumed that BPOP was more strongly activated by the oncogenic proteins (p53, β-catenin, BCL2, pAKT, and survivin) than osteochondroma; BPOP might be affected by oncogenic signaling of cellular proliferation and survival. Eventually, BPOP produced CEA, EMA, pan-K, and S-100, implying that BPOP might have higher potential for malignant transformation than osteochondroma. Since BPOP still showed no cellular atypia and expressed only low levels of embryonic chondrocyte proteins (CEA, EMA, pan-K, and S-100), it was also presumed that the growth of BPOP was primitive and immature, similar to the embryonal development of cartilage and bone, i.e., Meckel’s cartilage in mandible development, rather than to the progression of malignant transformation.

## Conclusions

The present study used IHC staining to compare a case of BPOP occurring at the lingual area of the right mandibular body with a case of representative osteochondroma occurring at the left mandibular condyle. Although the BPOP specimen showed no features of cellular atypia or malignant transformation, it did express more osteogenic proteins (BMP-2, BMP-4, RUNX2, OC, AP, OPG, RANKL CTGF, and bFGF) than osteochondroma. Furthermore, the perichondral spindle cells and marrow osteoblasts/fibroblasts of BPOP showed stronger immunoreaction of PCNA, p53, β-catenin, BCL2, pAKT, survivin, CEA, EMA, pan-K, and S-100 than the tumor cells of osteochondroma. Therefore, it was presumed that, similar to the embryonal osteochondroid tissues, BPOP might be activated by the osteogenic and oncogenic signalings and that this increased growth signaling may explain the rapid growth and high recurrence of BPOP.

### Consent

Written informed consent was obtained from the patient for publication of this manuscript and any accompanying images. A copy of the written consent is available for review by the Editor-in-Chief of this journal.
